# Intraosseous Orbital Cavernous Hemangioma with Frontal Extension and Dural Involvement

**DOI:** 10.7759/cureus.4823

**Published:** 2019-06-04

**Authors:** Asad S Akhter, Najib El Tecle, Georgios Alexopoulos, Gabriela Espinoza, Jeroen Coppens

**Affiliations:** 1 Neurosurgery, Ohio State University, Wexner Medical Center, Colombus, USA; 2 Neurosurgery, St. Louis University Hospital, St. Louis, USA; 3 Ophthalmology, St. Louis University Hospital, St. Louis, USA

**Keywords:** cavernous hemangioma, orbit, skull base, craniotomy, skull tumor, skull lesion, dura mater, dural involvement, case report

## Abstract

Primary intraosseous cavernous hemangiomas are rare skull lesions that are not typically known to involve the orbital bones or the dura. We describe a rare case of a fronto-orbital bone cavernous hemangioma with extension into the dura.

A 68-year-old female presented with a one-year history of diplopia with discomfort around her left orbit. Magnetic resonance images demonstrated a mass in the left frontal skull extending into the orbital rim. The patient underwent a craniotomy for tumor resection. Dural invasion was found intraoperatively. Gross total resection and reconstruction were achieved. On the postoperative follow-up, the patient was asymptomatic.

Primary calvarial intraosseous cavernous hemangiomas are most commonly located in the frontal and parietal bones. These lesions typically involve only the outer table of the skull. In lesions involving the orbit and dura, excision with cranioplasty can provide symptomatic relief with good cosmetic outcomes.

## Introduction

Primary intraosseous cavernous hemangiomas are rare, benign vascular lesions, accounting for 0.2% of bone tumors and 7% of skull tumors [1]. Although these lesions can occur anywhere in the skull, the most common locations are the frontal and parietal bones [2]. Involvement of the orbital bones by the hemangioma is exceedingly rare [3-4].

Intraosseous hemangiomas of the skull tend to involve the outer table of the skull and spare the inner table and dura matter. Intradural extension of a cavernous hemangioma has only been reported once in the literature [3]. A few case reports of involvement of the inner table have been described [1-2].

We present a unique and rare case of a fronto-orbital bone cavernous hemangioma with an extension of the hemangioma beyond the inner table and with the involvement of the dura.

## Case presentation

**Figure 1:**
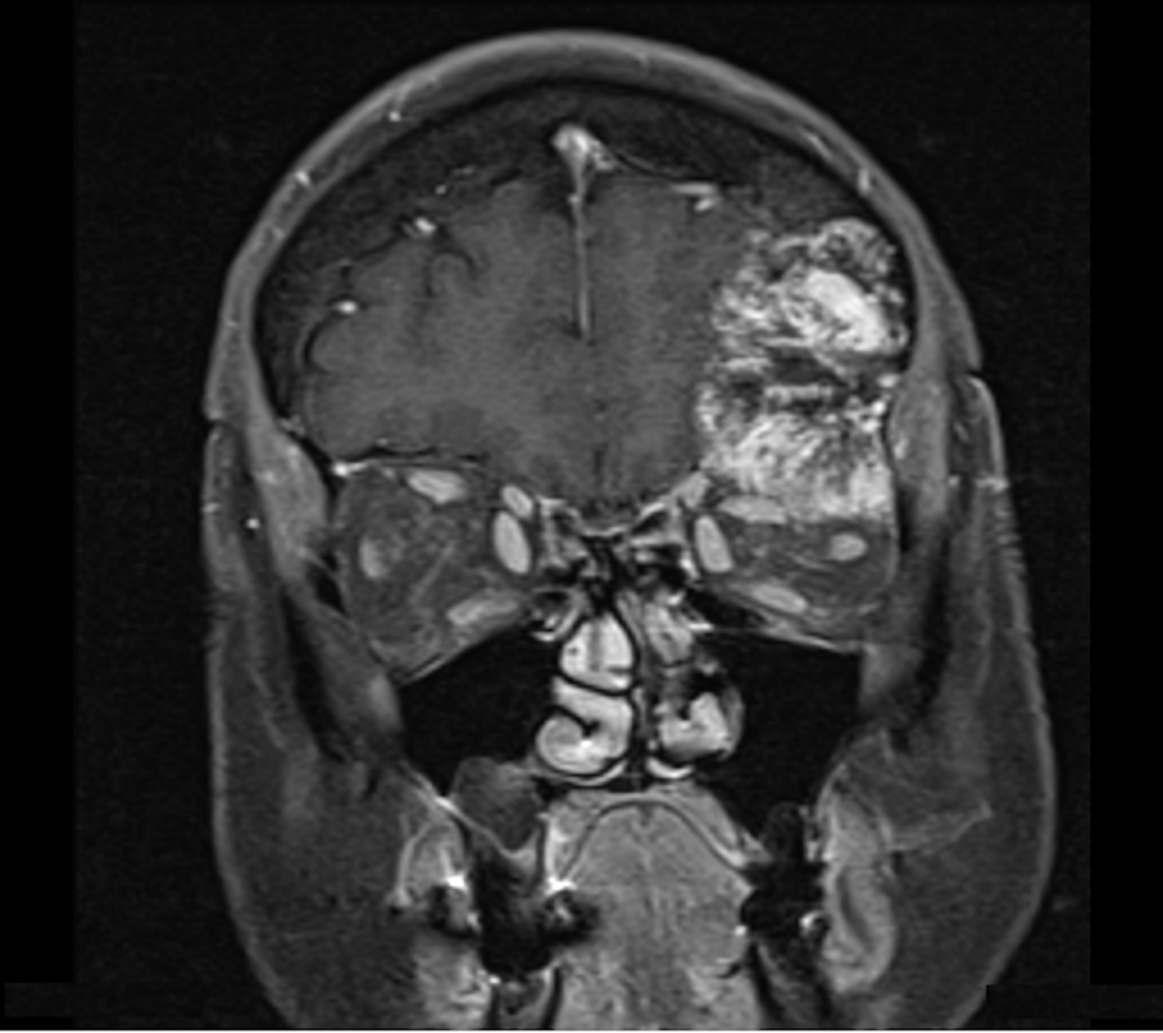
Preoperative coronal T1 contrast-enhanced magnetic resonance imaging

**Figure 2:**
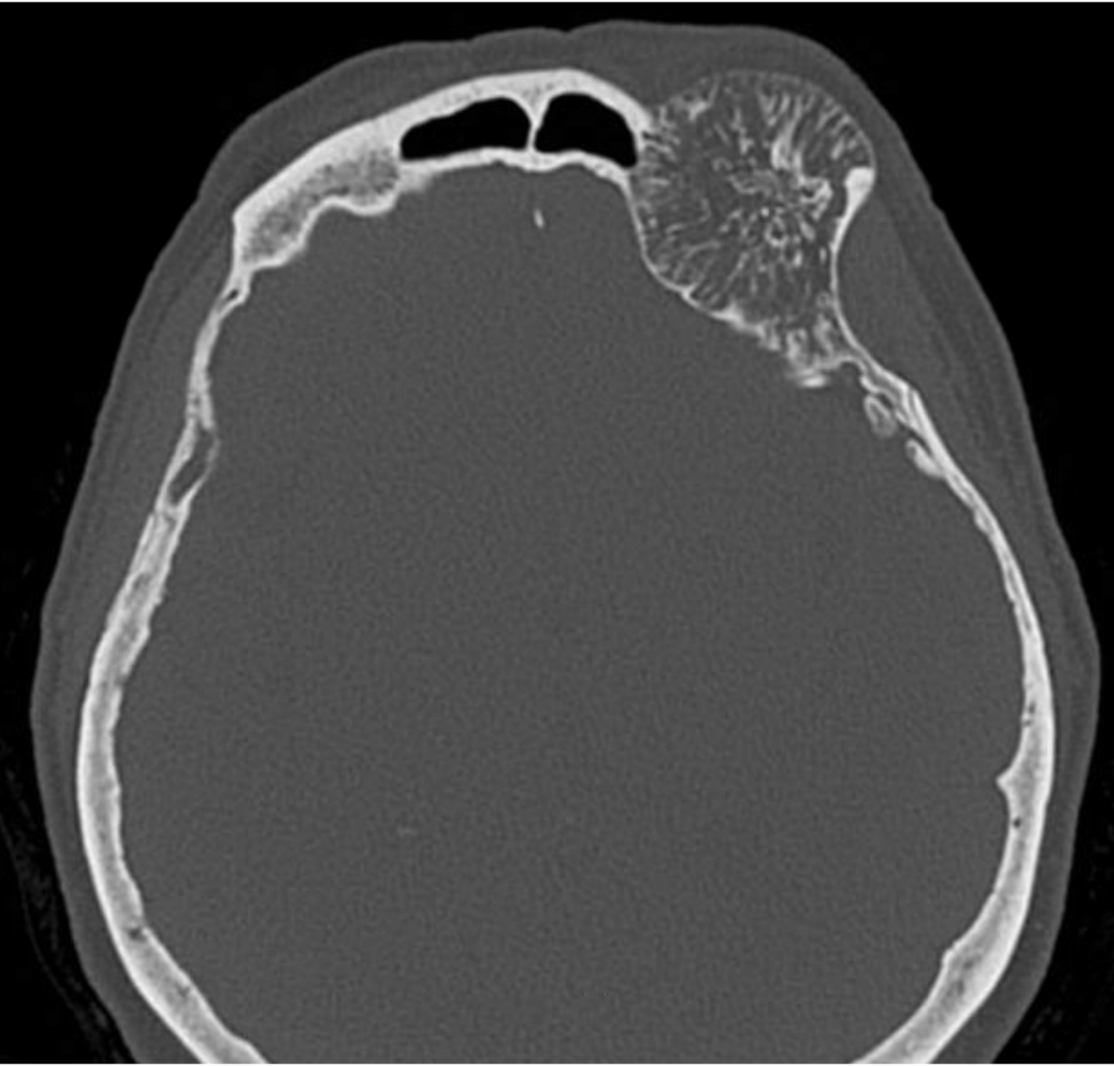
Preoperative axial computed tomography scan

A 68-year-old woman presented with a one-year history of diplopia associated with discomfort and pressure around her left orbit and forehead. Physical exam demonstrated proptosis with a 4 mm downward displacement of the left globe. The left inferior orbital rim was not readily palpable. The remainder of the exam was normal. Magnetic resonance imaging (MRI) demonstrated a 5.7 cm x 3.9 cm x 4.4 cm mass in the left frontal skull extending into the left orbital rim and into the left orbit with a displacement of the superior rectus muscle and downward displacement of the globe (Figure 1). The mass also indented the inferior left frontal lobe. A fine needle aspiration (FNA) of the soft tissue component in the left superior orbit was performed. Cytology report was compatible with a hematoma and was negative for a neoplasm. A computed tomography (CT) scan of the head without contrast demonstrated a well-circumscribed, expansile 5.3 cm x 3.7 cm x 4.5 cm mass with coarse internal trabeculations and sclerotic margins in the supraorbital left frontal bone, consistent with an intraosseous hemangioma (Figure 2). A nuclear medicine bone scan demonstrated a circular area with moderate to intense Tc-99m methylene diphosphonate (MDP) uptake in the left supraorbital frontal skull lesion, consistent with a hemangioma. The patient wished to proceed with surgery. 

**Figure 3:**
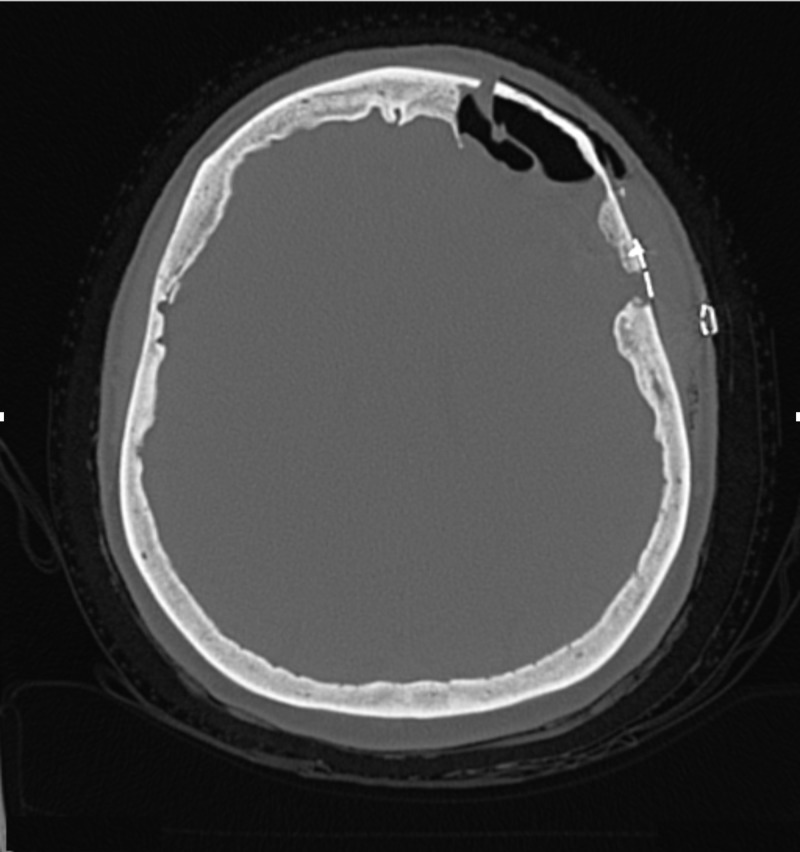
Postoperative computed tomography of the head

The patient underwent a left orbito-zygomatic (OZ) craniotomy for resection of the involved bone and subsequent reconstruction of the orbital walls. The patient was placed in a Mayfield headrest and stereotactic registration was accomplished. A standard OZ approach was performed. After drilling the first access burr hole, it was noted that the dura was not preserved and could not be separated from the inner table. Two additional burr holes and cuts involving the zygoma and orbit were made for access. A side cutting drill was used to turn a bone flap but this could not be mobilized freely. An osteotome had to be used to remove the outer table with some part of the middle table. Further drilling of the remaining hemangioma was performed with a small cutting burr, followed by a diamond burr under the microscope. The bony tumor was found to extend both extra- and intradurally. Drilling of the abnormal bone was continued medially to the edge of the frontal sinus. The dura was preserved when feasible, but significant areas of dura were not preserved. The dural defect was closed with a suturable dural substitute (Durerepair®) (Medtronic, Minneapolis, MN, USA) and some tacking at the bony edges where a watertight closure was not feasible. Upon complete resection, a piece of porous polyethylene was cut and contoured (Medpore®) (Stryker, Kalamazoo, MI, USA) to recreate the superior orbital rim and the roof of the orbit. The porous polyethylene was fixated laterally to the zygoma and medially to the frontal bone with standard cranial screws. The closure was done using the remainder of the bone flap, which was attached with standard screws and plates (OsteoMed, Addison, TX, USA) after drilling the involved portion. A postoperative CT scan showed complete resection of the hemangioma (Figure 3).

**Figure 4:**
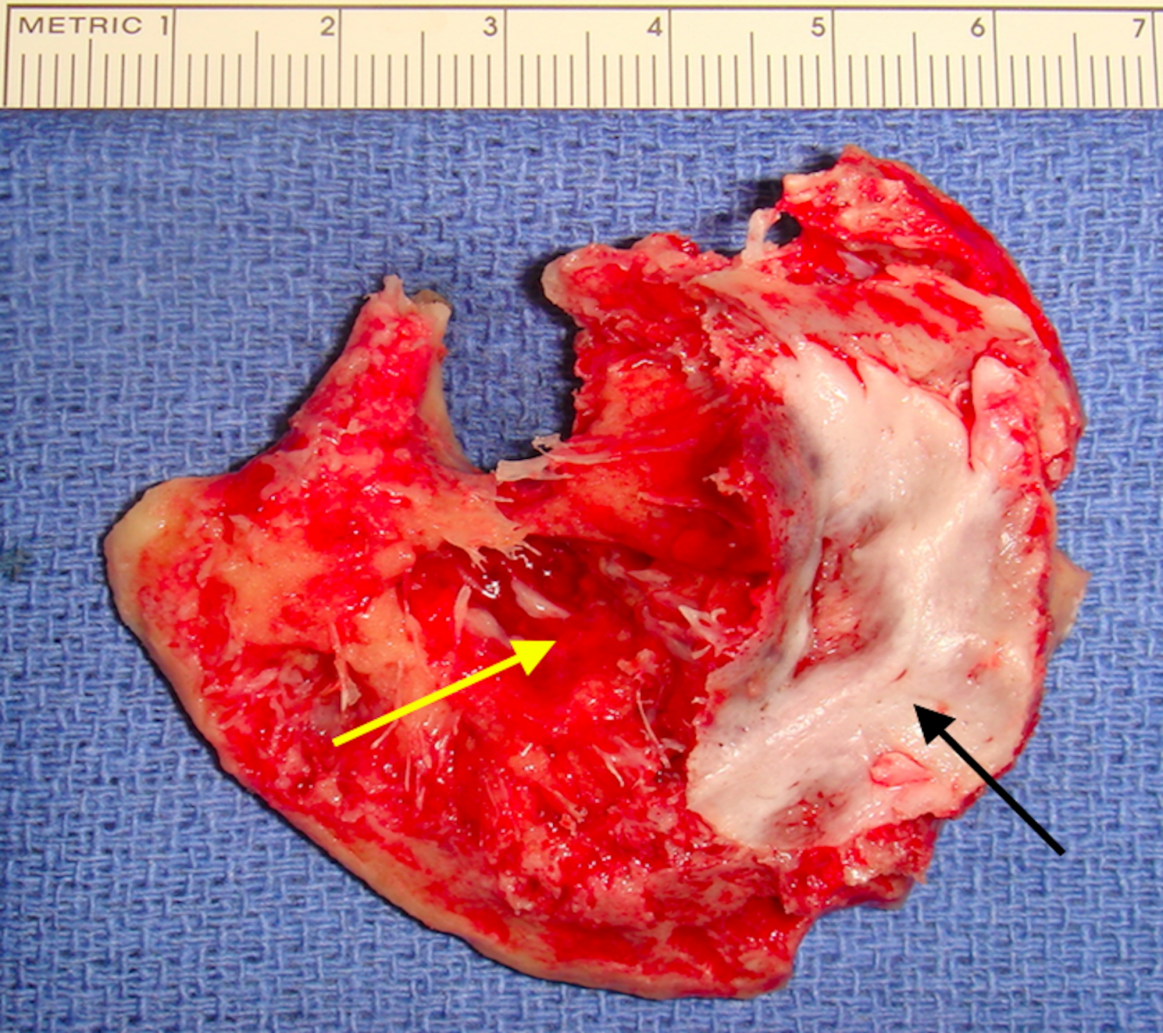
Intraoperative picture demonstrating bony involvement. The yellow arrow shows red discoloration of the bone and represents involvement of the inner table of the skull. The black arrow shows an intact inner table.

Gross examination of the left frontal bone specimens sent during surgery demonstrated red-white bony tissue (Figure 4). Microscopic examination described bone medulla with abnormally large crowded capillaries and venules without atypical endothelium, consistent with a benign cavernous hemangioma. 

**Figure 5:**
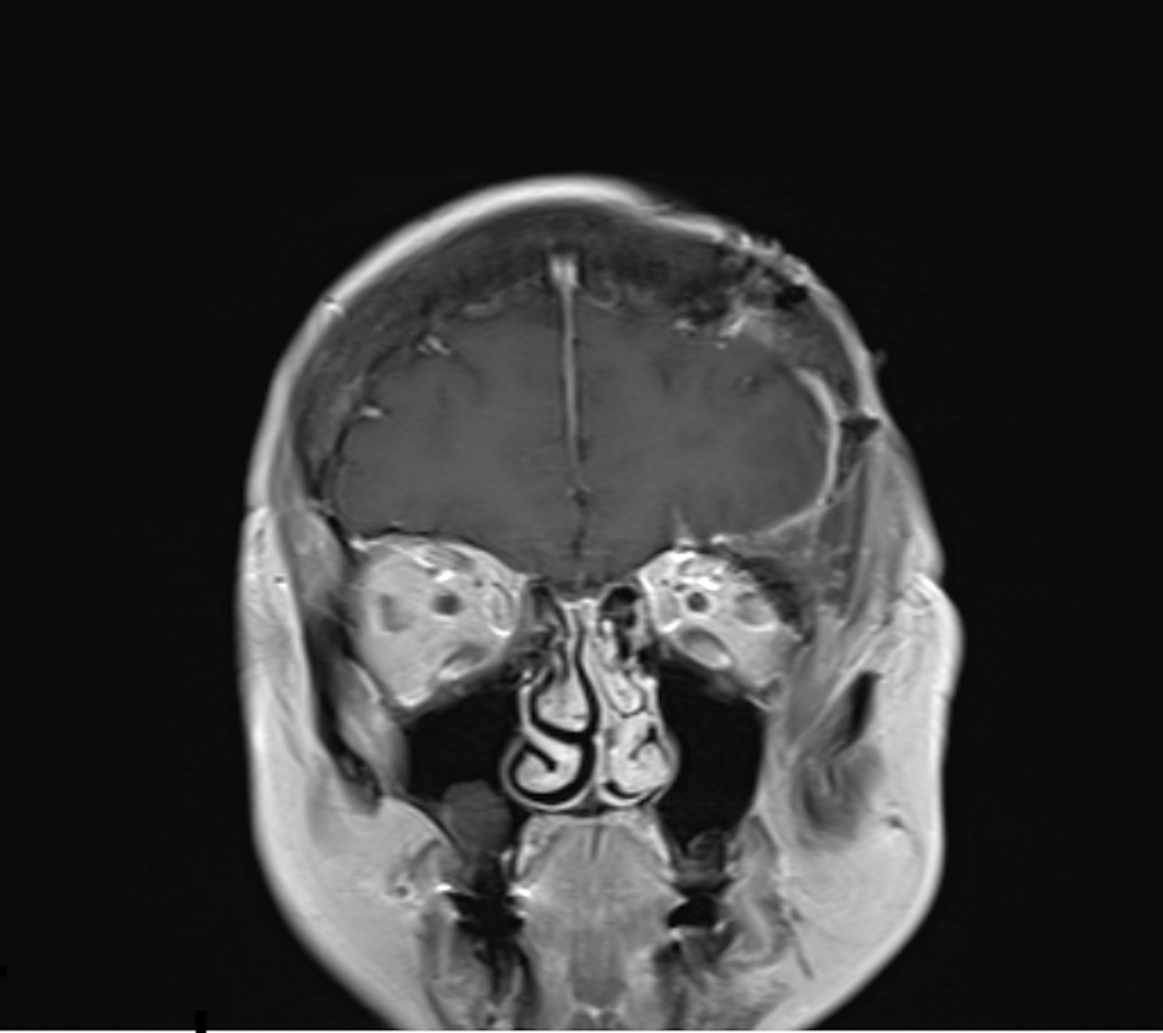
Postoperative coronal T1 contrast-enhanced magnetic resonance imaging demonstrating complete resection of the tumor.

The postoperative MRI demonstrated complete resection of the lesion (Figure 5). The patient was discharged on postoperative Day 3. On Day 10 follow-up, the diplopia had resolved. Physical examination demonstrated improving proptosis. At her one month follow-up, the patient continued to have no complaints of diplopia, as well as no complaints of pain or the sensation of pressure and fullness behind the left orbit. The patient remained asymptomatic at four months of follow-up with stable imaging.

## Discussion

Hemangiomas are normally benign, asymptomatic tumors of blood vessels. They most commonly occur in females with a 3:2 ratio. The mean age of presentation is 46 years [1, 4-6]. 

Three histological subtypes of hemangiomas (based on the size of vascular channels) have been described: cavernous, capillary, and mixed. Vessels of cavernous hemangiomas are large and closely clustered, in contrast to vessels of the capillary subtype, which are thin-walled and separated by normal bone tissue [7-11]. Most calvarial hemangiomas are cavernous, as seen in this case, while vertebral hemangiomas are capillary [8, 10]. Though previous trauma has been suggested as a possible etiology, definitive pathogenesis is unknown [12-13]. 

Intraosseous hemangiomas of the orbital bones are exceedingly uncommon. A total of 51 cases have been reported in the literature, and they are usually confined to the orbit [2, 7]. They most commonly present as an enlarging mass followed by pain [12, 14]. Diplopia as a presenting symptom has been reported in 13% of cases of orbital hemangiomas. 

Intracranial involvement of an orbital hemangioma has been reported in five other cases to date [2, 7]. The patients with such orbital hemangiomas with intracranial involvement all presented with proptosis (Table 1). Symptoms were present for an average of 18 months in the adult population. The cranial involvement affects the frontal bones in 66.7% and sphenoid bones in 33.3% of cases. Cavernous hemangiomas were observed in 66.7% of cases. All cases were treated with craniotomy, and cranioplasty was required in about 33.3% of cases, as in this case.

**Table 1:** Intraosseous orbital hemangiomas with intracranial extension

Year	Author	Age/Sex	Affected Bones	Main Clinical Findings	Duration of symptoms	Pathology	Surgery
1997	Sweet et al. [15]	8 F	Sphenoid	Proptosis, reduced vision, ophthalmoplegia	Acute (1 day)	Capillary	Craniotomy
1999	Slaba et al. [16]	55 M	Sphenoid	Proptosis, reduced vision	3 years	Cavernous	Craniotomy
2005	Shah et al. [10]	39 M	Frontal	Ptosis, ophthalmoplegia	1 year	Capillary	Craniotomy
2013	Gupta et al. [2]	49 M	Frontal	Midline frontal swelling, headache	2 years	Cavernous	Craniectomy with titanium cranioplasty
2013	Gupta et al. [2]	59 M	Frontal	Proptosis, inferior globe displacement	6 months	Cavernous	Craniectomy (type of cranioplasty not specified)

Cavernous hemangiomas of the calvarium usually originate from vessels in the diploic space with the erosion of the outer table and little to no involvement of the inner table [8]. Involvement of the inner table is rare, and transdural invasion has not been reported in detail in the literature. Involvement of the inner table or dura has been reported in two cases during resection of an elective calvarial hemangioma by direct observation [5, 13]. Another form of dural involvement has been noted in two other cases where the hemangioma hemorrhaged and was associated with a subdural or epidural hematoma [1, 11]. Nasi et al. reported a case of a calvarial cavernous hemangioma of the frontal bone with extensive intradural involvement [1]. Its resection also required cranioplasty. Only one other case of a combined orbital/intracranial cavernous hemangioma has been described by Shah et al. [10]. Their case showed the intracranial involvement to be restricted to the anterior skull base. Our case showed the involved dura to affect the frontal convexity and the dura was intact at the level of the orbital wall.

The classic characteristic appearance of the hemangioma on CT shows a “honeycomb” or “sunburst” pattern with fine, radiating, and reticulated lines or trabeculae [12]. MRI of these lesions shows intermediate to high T1 signal intensity and high heterogeneous T2 signal intensity [9]. There is an extensive differential diagnosis for intradiploic skull masses, including metastatic diseases, meningiomas, sarcomas, dermoid tumors, fibrous dysplasia, Paget disease, etc. [14]. MRI and CT findings, therefore, may not be pathognomonic or sufficient to make a definite preoperative diagnosis. As such, confirmatory diagnosis is made via surgery and biopsy [9]. Our patient presented with imaging on CT and MRI that was consistent with a hemangioma (Figures 1-2).

Treatment depends on the presenting signs and symptoms of the lesion or on its behavior on sequential imaging. Primary intraosseous hemangiomas typically are asymptomatic and follow a benign course. Surgery is reserved for patients with mass effect, as was the case for our patient. Other indications are hemorrhage or cosmetic concerns [12]. Surgical resection is the preferred treatment for orbital intraosseous cavernous hemangiomas [4]. Cases involving the orbit with a cranial extension may require skull base approaches and a combination of cranioplasty techniques may be necessary for repair.

## Conclusions

We present an unusual case of an orbital intraosseous cavernous hemangioma located at the junction to the inferior frontal bone. The patient was found to have some involvement of the inner table, as well as the dura. A skull base approach, combined with cranioplasty, resulted in resolution of symptoms with a good cosmetic outcome.
